# Identification of neutrophil extracellular trap-related biomarkers in ulcerative colitis based on bioinformatics and machine learning

**DOI:** 10.3389/fgene.2025.1589999

**Published:** 2025-06-20

**Authors:** Jiao Li, Yupei Liu, Zhiyi Sun, Suqi Zeng, Caisong Zheng

**Affiliations:** ^1^ Department of Gastroenterology, Renmin Hospital of Wuhan University, Wuhan, China; ^2^ Department of Biostatistics, School of Public Health, University of Michigan, Ann Arbor, MI, United States; ^3^ Department of Gastrointestinal Surgery, People’s Hospital of Macheng City, Macheng, China

**Keywords:** inflammatory bowel disease, ulcerative colitis, neutrophil extracellular traps, bioinformatics, machine learning

## Abstract

**Background:**

The incidence of ulcerative colitis (UC) is rapidly increasing worldwide, but existing therapeutics are limited. Neutrophil extracellular traps (NETs), which have been associated with the development of various autoimmune diseases, may serve as a novel therapeutic target for UC treatment.

**Methods:**

Bioinformatics analysis was performed to investigate UC-related datasets downloaded from the GEO database, including GSE87466, GSE75214, and GSE206285. Differentially expressed genes (DEGs) related to NETs in UC patients and healthy controls were identified using Limma R package and WGCNA, followed by functional enrichment analysis. To identify potential diagnostic biomarkers, we applied the Least Absolute Shrinkage and Selection Operator (LASSO), Support Vector Machine-Recursive Feature Elimination (SVM-RFE) model, and Random Forest (RF) algorithm, and constructed Receiver Operating Characteristic (ROC) curves to evaluate accuracy. Additionally, immune infiltration analysis was conducted to identify immune cells potentially involved in the regulation of NETs. Finally, the expression of core genes in patients was validated using Quantitative real-time PCR (qRT-PCR), and potential therapeutic drugs for UC were explored through drug target databases.

**Result:**

Differential analysis of transcriptomic sequencing data from UC samples identified 29 DEGs related to NETs. Enrichment analysis showed that these genes primarily mediate UC-related damage through biological functions such as leukocyte activation, migration, immune receptor activity, and the IL-17 signaling pathway. Three machine learning algorithms successfully identified core NETs-related genes in UC (IL1B, MMP9 and DYSF). According to ROC analysis, all three demonstrated excellent diagnostic efficacy. Additionally, Immune infiltration analysis revealed that the expression of these core genes was closely associated with neutrophils infiltration and CD4^+^ memory T cell activation, and negatively associated with M2 macrophage infiltration. qRT-PCR showed that the core genes were significantly overexpressed in UC patients. Gevokizumab, canakinumab and carboxylated glucosamine were predicted as potential therapeutic drugs for UC.

**Conclusion:**

By combining three machine learning algorithms and bioinformatics, this research identified three hub genes that could serve as novel targets for the diagnosis and therapy of UC, which may provide valuable insights into the mechanism of NETs in UC and potential related therapies.

## Introduction

Ulcerative colitis (UC) is a chronic and relapsing inflammatory bowel disease that primarily affects the mucosa of the colon and rectum, presenting with symptoms such as diarrhea, mucus-purulent bloody stools, and possible extra-intestinal manifestations (Gros and Kaplan, 2023). The etiology of UC is complex and multifactorial, involving genetic susceptibility (Chen et al., 2024a), immune system abnormalities (Baars et al., 2024), dysbiosis of the (Yan et al., 2024), and dysfunction of the intestinal epithelial barrier (Neurath et al., 2025). Among the mechanisms underlying UC, abnormal mucosal immune responses and inflammation are key pathological features, and neutrophils playing a crucial role in maintaining intestinal immune homeostasis (Noviello et al., 2021).

Neutrophils are an essential component of the innate immune system, defending against microorganisms through phagocytosis, degranulation, and the release of extracellular traps (NETs), which enhance immune defense (Brinkmann et al., 2004). During the pathogenesis of inflammatory bowel disease (IBD), the formation of NETs can activate the production of various pro-inflammatory factors, such as IL-1β, TNF-α, and IL-17A. Furthermore, NETs found in inflamed intestinal tissues of IBD are enriched with myeloperoxidase, lactoferrin, and calprotectin, which collaboratively contribute to the progression of IBD (Zhou et al., 2018). NETs also disrupt the intestinal epithelial barrier by promoting the breakdown of cell-cell junctions and inducing apoptosis in epithelial cells, leading to increased intestinal permeability to luminal antigens. Additionally, NETs promote intestinal inflammation by mediating the enhanced production and release of inflammatory mediators by resident immune cells and by degrading extracellular matrix components, thereby disrupting connective tissue (Li et al., 2020; Wang H. et al., 2024). Abnormal accumulation of NETs and their failure to be effectively degraded may worsen tissue damage in the gut, contributing to disease persistence and progression. Investigating the role of NETs in UC could provide new insights and potential therapeutic targets for the diagnosis and treatment of UC in the future.

**FIGURE 1:**
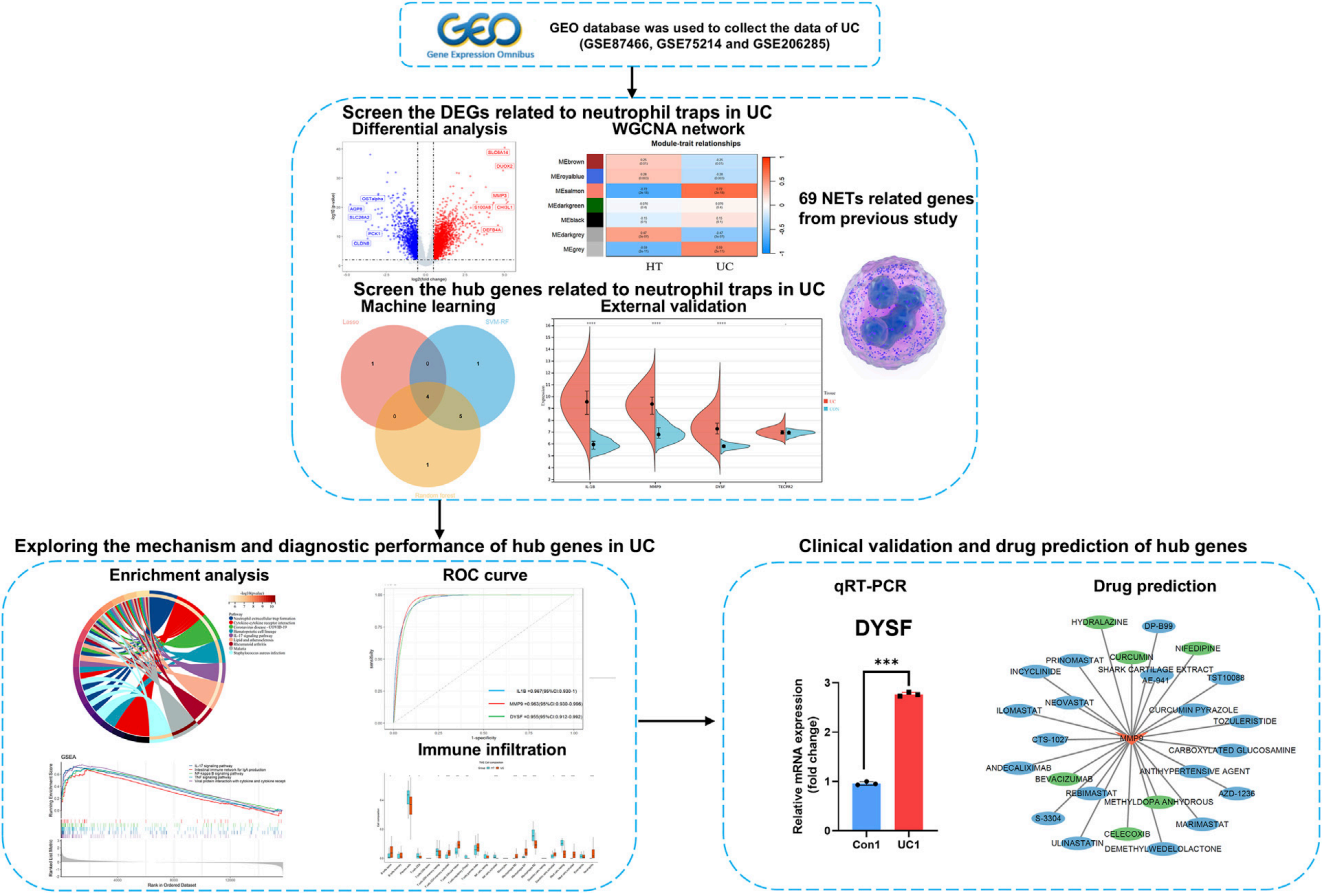
Roadmap of the main research ideas in this article.

In this study, based on UC-related datasets in the GEO database and NETs related genes (NRGs) collected in the literature, differentially expressed genes related to neutrophil extracellular traps (DEONRGs) was obtained after differential and weighted gene co-expression network analysis (WGCNA). Subsequently, enrichment analysis to explore the molecular mechanisms and biological functions of DEONRGs. core genes related to NETs were identified by machine learning models and external data. The potential of these core genes as diagnostic biomarkers for UC was assessed using Receiver Operating Characteristic (ROC) curves. Additionally, immune cell infiltration and biological pathways associated with core genes were investigated through immune infiltration analysis and GSEA. Finally, the expression of core genes in patients was validated using Quantitative real-time PCR (qRT-PCR), and potential therapeutic drugs for UC were explored through drug target databases. This study provides an in-depth investigation that enhances our understanding of the complex interactions of NETs in the pathogenesis of UC. The detailed workflow of the analysis is shown in Figure 1.

## Methods

### Data source and processing

All RNA-seq datasets included in this study were obtained from the Gene Expression Omnibus (GEO) database (http://www.ncbi.nlm.nih.gov/geo), using “inflammatory bowel disease” as a keyword to search for relevant expression datasets. Three independent datasets were selected, including GSE87466 as the training set and GSE75214 and GSE206285 as validation sets (Supplementary Table 1). After importing the data into R software (version 4.3.2, https://www.r-project.org/), Probes were converted to gene symbols according to the platform annotation information of the normalized data. probes without corresponding gene symbols were excluded to maintain data integrity. For probes mapping to the same gene, the mean expression value was used as the final expression value to ensure accuracy and consistency. The expression profiles were normalized using the Normalize Between Arrays function from the “limma” package, and the normalized data were subsequently used for further analysis (Ritchie et al., 2015). Additionally, the 69 initial NETs biomarkers included in this study were sourced from previous research (Zhang et al., 2022a) (Supplementary Table 2).

### DEGs and weighted co-expression network

To identify differentially expressed genes (DEGs) associated with NETs, we utilized 2 R packages, “GEOquery” and “Limma,” to retrieve the raw data and perform differential expression analysis. The “Limma” package was used to compare gene expression profiles across different groups, with thresholds set at a *p*-value <0.01 and |logFC| > 1 to identify DEGs between the case and control groups (Ritchie et al., 2015). Subsequently, we employed the “WGCNA” package for WGCNA on the training dataset expression data (Langfelder and Horvath, 2008). To ensure the co-expression network conforms to a scale-free distribution, the soft threshold power was determined using the pickSoftThreshold function. The dynamic tree cut method was applied to define separate modules, each containing at least 30 genes. To consolidate similar modules, a mergeCutHeight of 0.3 was set. The association between the identified modules and UC was further explored. The module most closely related to UC, with a correlation coefficient exceeding 0.5 (*p*-value <0.05), was isolated. Modules with a |MM| greater than 0.8 and |GS| exceeding 0.4 were deemed critical. Module membership (MM) quantifies the relationship between genes and the module, while gene significance (GS) measures the correlation between genes and the trait. Finally, a Venn diagram was generated (https://www.bic.ac.cn/EVenn/#/) to intersect the DEGs with the final modules and NETs-related genes, which led to the identification of 29 DEONRGs between UC and control samples.

### PPI network construction

To explore the interactions between 29 DEONRGs, we constructed a Protein-Protein Interaction (PPI) network (Szklarczyk et al., 2023). Specifically, the PPI network was built using protein interaction data from the STRING database (https://cn.string-db.org/), Interactions with a composite score surpassing 0.4 were deemed statistically significant.

### Enrichment analysis

Gene Ontology (GO) enrichment analysis (http://www.geneontology.org) and Kyoto Encyclopedia of Genes and Genomes (KEGG) enrichment analysis (www.genome.jp/kegg/) were employed to determine the biological functions of the genes (Kanehisa et al., 2017; Yu et al., 2012). GO terms consist of three categories: Biological Process (BP), Cellular Component (CC), and Molecular Function (MF). Significant pathways with a *p*-value <0.05 were selected for further analysis.

### Screening biomarkers by machine learning models

To identify key NETs-related biomarkers, we utilized three machine learning algorithms: Least Absolute Shrinkage and Selection Operator (LASSO) regression, Support Vector Machine-Recursive Feature Elimination (SVM-RFE), and Random Forest (RF). Prior to applying LASSO, SVM-RFE, and RF, the Synthetic Minority Over-sampling Technique from the ‘smotefamily’ package was employed to balance the imbalanced data (Bunkhumpornpat et al., 2024). This procedure aimed to mitigate any potential bias toward the majority class and uphold the integrity of the analysis. Following this, the createDataPartition function from the ‘caret’ package was utilized to divide the balanced data into training and test sets, with an 8:2 ratio. This division enables the model to be trained on one subset and evaluated on an independent test subset, ensuring a fair evaluation of the algorithm’s performance. LASSO is a widely used regression method that selects variables to improve prediction accuracy. It was implemented using the “glmnet” R package (version 4.1). We selected the optimal λ value by cross validation and removed genes exhibiting multicollinearity to reduce potential bias (Kang et al., 2021). The SVM-RFE was implemented using the “e1071”R package (version 4.1). We optimized the C and γ parameters through 10-fold cross-validation and grid search to select the best configuration in training sets (Zhang et al., 2022b). The RF algorithm, a supervised classification method based on decision trees, was implemented using the “randomForest” R package (version 4.7). Similarly, we evaluated the error rate across tree counts ranging from 1 to 500 and determined the optimal number of trees by performing 10-fold cross-validation in training set to select the configuration with the lowest error rate in training sets (Schonnagel et al., 2024). Additionally, we measured the feature importance scores of each gene, identifying candidate hub genes with an importance value greater than 1. Finally, The validation set is used to construct the confusion matrix of the machine learning models and output the parameters including accuracy, precision, recall, F1 score, and AUC to evaluate the performance of different models (Rainio et al., 2024).

### External validation and diagnostic performance of core genes

To validate our findings, we assessed the expression of the core genes using two external datasets, GSE75214 and GSE206285. Genes that exhibited differential expression in both datasets were identified as core genes. Subsequently, the ‘pROC’ package was utilized to generate the receiver operating characteristic (ROC) curve, evaluating the ability of the hub genes to distinguish between UC patients and healthy individuals across all datasets (Robin et al., 2011).

### Gene set enrichment analysis

To investigate the relationship between hub genes and signaling pathways, and to further elucidate the key role of DEONRGs in the pathogenesis of UC, we divided the samples into high-expression and low-expression groups based on the average expression levels of the hub genes. Gene set enrichment analysis (GSEA) was then performed between these two subgroups (Chang et al., 2024). Gene sets showing enrichment with a nominal *p*-value of <0.05, |normalized enrichment score (NES)| > 1, and a false discovery rate (FDR) *q*-value <0.25 were classified as statistically significant.

### Immune infiltration of core genes

To further explore the potential relationship between core genes and immune cell populations, immune infiltration analysis was performed using the R package “CIBERSORT.” Subsequently, Spearman’s correlation analysis was conducted to examine the relationship between the expression of diagnostic biomarkers and the abundance of 22 different immune cell types (Newman et al., 2015).

### Quantitative real-time polymerase chain reaction

Quantitative Real-Time Polymerase Chain Reaction (qRT-PCR) was performed to determine the NETs expression profile in UC patients. Blood samples were collected from three patients with active UC, diagnosed based on confirmed pathological biopsy, as well as from three age-matched healthy controls. The average age of all samples was 38 ± 4.2 years, and no significant age difference was observed between the two groups (*P* > 0.05). Informed consent was obtained from all participants. This study was approved by the Ethics Committee of Renmin Hospital of Wuhan University (Ethics No: 2022K‐K265 (Y01)). Total RNA was extracted from six blood samples using TRIzol reagent (Ambion, Austin, USA). cDNA was synthesized from total RNA using a first-strand cDNA synthesis kit (Servicebio, Wuhan, China). qRT-PCR was performed using 2xUniversal Blue SYBR Green qPCR Master Mix (Servicebio, Wuhan, China). All experiments were conducted according to the manufacturer’s instructions. Primer sequences for PCR were designed based on primer length (17–25 bp), Tm value (58°C–60°C), GC content (40%–60%), and amplicon size (100–200 bp) (Supplementary Table 3). GAPDH was used as an internal reference gene. Gene expression was calculated using the 2^−ΔΔCq^ method (Livak and Schmittgen, 2001).

### Prediction of potential drugs

Based on the identified diagnostic UC biomarkers, potential drugs for UC treatment were predicted using the DGIdb database (https://www.dgidb.org/) (Cannon et al., 2024). The biomarker-compound interaction network was visualized using Cytoscape software (version 3.9.1) (Franz et al., 2023).

### Statistical analysis

Differences between the two groups were analyzed using the unpaired Student’s t-test and the Wilcoxon rank-sum test. Pearson or Spearman correlation analysis was used to assess the relationships between variables. Statistical analysis and data visualization were performed using GraphPad Prism 8.0.2 and R 4.3.2 software. Unless otherwise stated, differences were considered statistically significant when *p* < 0.05 (*p < 0.05, **p < 0.01, ***p < 0.001).

## Results

### Screening DEGs in UC

**FIGURE 2:**
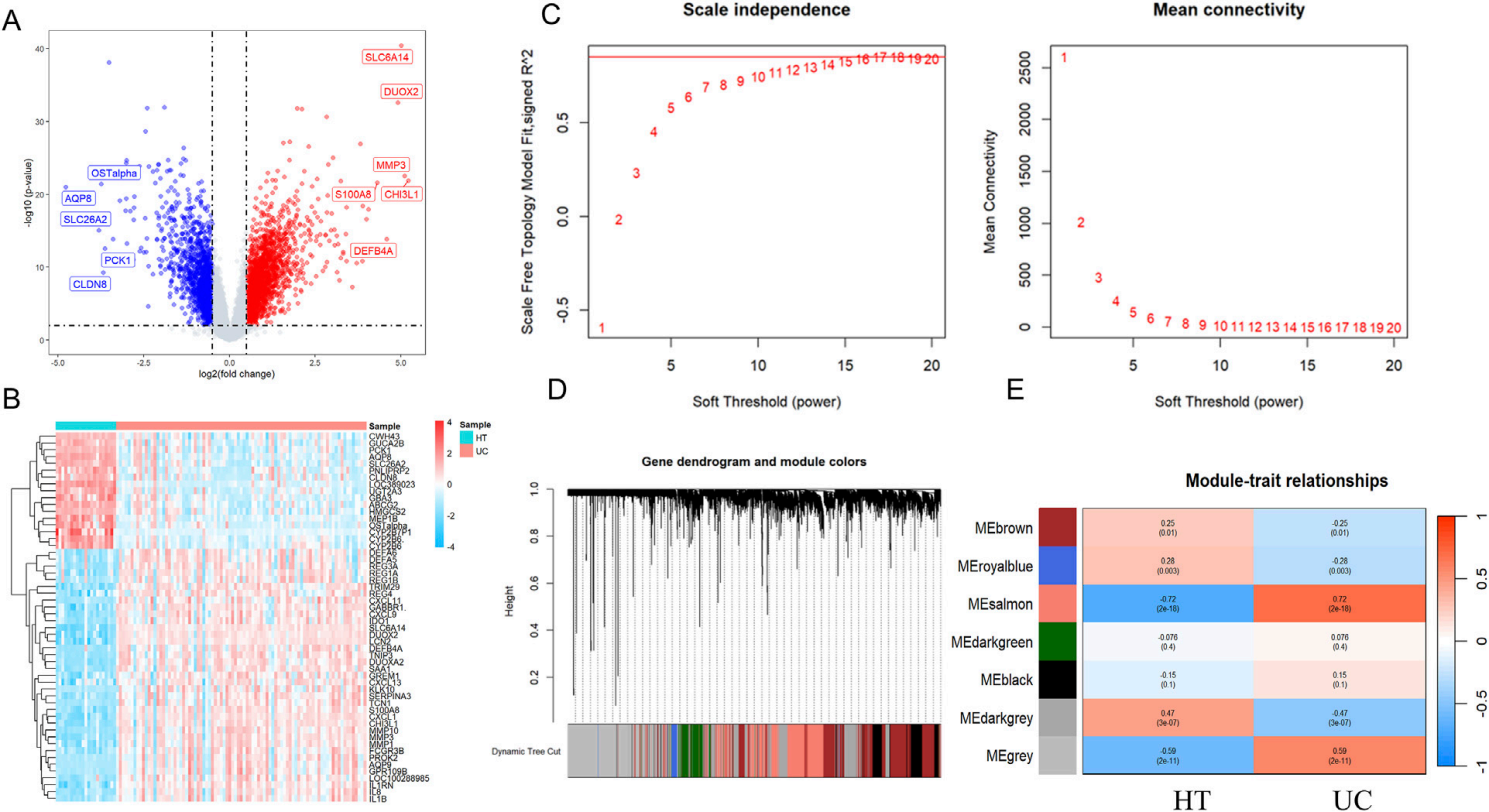
Identification of differentially expressed genes and trait-related gene modules in UC. Volcano plots **(A)** and heatmap **(B)** illustrating DEGs in UC datasets. Each point represents a gene, with red and blue indicating significantly upregulated and downregulated genes, respectively, based on thresholds of |lgFC| > 1 and *p*-value <0.01. Grey dots represent non-significant genes. **(C)** Scale independence and mean connectivity in the GSE87466. **(D)** Gene dendrogram and modules after merging in the GSE87466. **(E)** Heatmaps showing module–trait relationships based on WGCNA in UC. Each row represents a co-expression module labeled by color, and each column represents a clinical trait (Healthy Control or UC). The values within each cell represent the Pearson correlation coefficient between the module eigengene and the trait, with the corresponding *p*-value shown in parentheses. Modules with strong positive or negative correlations are highlighted in red and blue, respectively, indicating potential trait relevance. WGCNA, Weighted gene co-expression network analysis; DEGs, differentially expressed genes.

DEGs from 87 UC samples and 21 control samples in the GSE87466 dataset were rigorously analyzed using the “limma” package in R for statistical analysis. Transcriptomic analysis identified 3,327 DEGs, including 1,876 upregulated and 1,350 downregulated genes (Figure 2A). A heatmap displaying the top 50 upregulated and downregulated DEGs, clustered by sample, is shown in Figure 2B.

As shown in Figure 2C, we constructed a sample clustering tree and corresponding clinical feature heatmap using the WGCNA package. After applying hierarchical clustering and dynamic tree cutting functions to identify the samples without outliers, we selected the top 10,000 genes based on expression levels from 108 samples for subsequent WGCNA analysis. To determine the appropriate soft-thresholding power for WGCNA, we assessed scale independence and average connectivity. Based on a correlation coefficient threshold of 0.85, we selected the ideal soft-thresholding power of 17 from the scale-free topology fit index plot and constructed the topological overlap matrix (TOM) accordingly. To identify modules associated with UC clinical features, we performed hierarchical clustering of the dendrograms of all DEGs using the corresponding dissimilarity (1-TOM). After dynamic tree pruning and average hierarchical clustering (Figure 2D), seven major modules were identified. Modules that exhibited strong correlations with clinical features typically have significant and specific biological relevance. We examined the Pearson correlation coefficients between the modules and sample characteristics. Among them, the salmon module (Correlation: 0.72, *p*-value: 2e-18) showed the strongest association with UC (Figure 2E). To further investigate the relationship between the salmon module and gene significance (GS), an in-depth analysis was conducted. The salmon module was found to have a correlation of 0.61 (*p*-value: 1.4e-187) with gene significance (Supplementary Figure 1A). This module contains 1,837 UC-related genes, which will be further exploration of NRGs in UC.

### PPI network and enrichment analysis of DEONRGs

**FIGURE 3:**
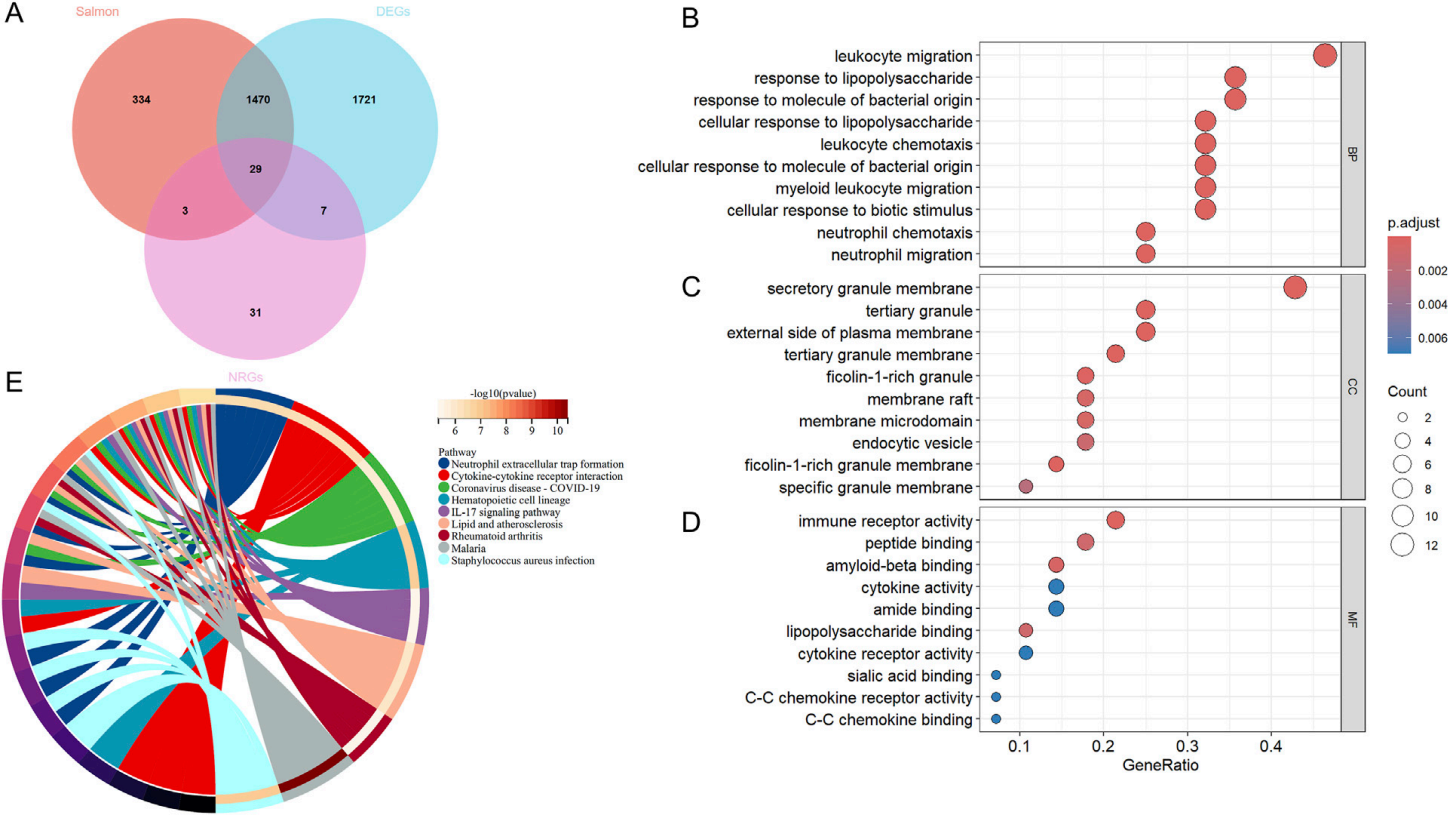
GO and KEGG Enrichment Analysis of DEONRGs in UC. **(A)** Venn diagram of DEONRGs in UC identified through WGCNA network analysis, Differential analysis and overlap with NRGs; **(B–D)** Dot plots showing GO enrichment analysis of DEONRGs; **(E)** KEGG pathway enrichment analysis of DEONRGs. An adjusted *p*-value <0.05 was considered statistically significant. The ordinate represents the enriched terms, and the abscissa represents the proportion of genes involved in each term. The size of the dots indicates the number of genes, while the color of the dots reflects the *p*-value. WGCNA, Weighted gene co-expression network analysis; DEONRGs: differentially expressed genes related to neutrophil extracellular traps; BP: Biological Process; CC, Cellular Component; MF, Molecular Function; GO, Gene Ontology; KEGG, Kyoto Encyclopedia of Genes and Genomes.

We performed a cross-analysis of NRGs with DEGs, resulting in 29 DEONRGs, as depicted in the Venn diagram (Figure 3A). To further elucidate the potential relationships of DEONRGs in UC, we conducted a PPI network analysis using STRING, incorporating 29 genes into the network. The resulting network contained 29 nodes and 157 edges (p < 1.0e‐16), with the depth and size of the nodes indicating the number of connections for each gene (Supplementary Figure 1B).

Furthermore, to explore the pathways involved in the intersecting genes, we created a dot plots for the key pathways identified through GO enrichment analysis and KEGG enrichment analysis, including BP (Figure 3B), MF (Figure 3C), and CC (Figure 3D). We also displayed the important pathways in network form (Figure 3E). In terms of biological processes (GO enrichment), these genes were primarily enriched in immune-related processes, such as inflammation, leukocyte and neutrophil chemotaxis, and chemokine-mediated signaling pathways. For molecular functions and cellular components, these DEGs were enriched in immune receptor activity, cytokine activity and secretory granule membrane. In the KEGG pathway enrichment analysis, besides the significant enrichment in NETs formation, the DEONRGs were also associated with cytokine-cytokine receptor interactions, the IL-17 signaling pathway, and pathways related to Coronavirus disease - COVID-19, among others. This suggests that DEONRGs may primarily enhance immune cell activity and drive the migration of leukocytes and neutrophils through the IL-17 signaling pathway and cytokine receptor interactions, contributing to the progression of UC and other various diseases.

### Core gene selection of DEONRGs by machine learning

**FIGURE 4:**
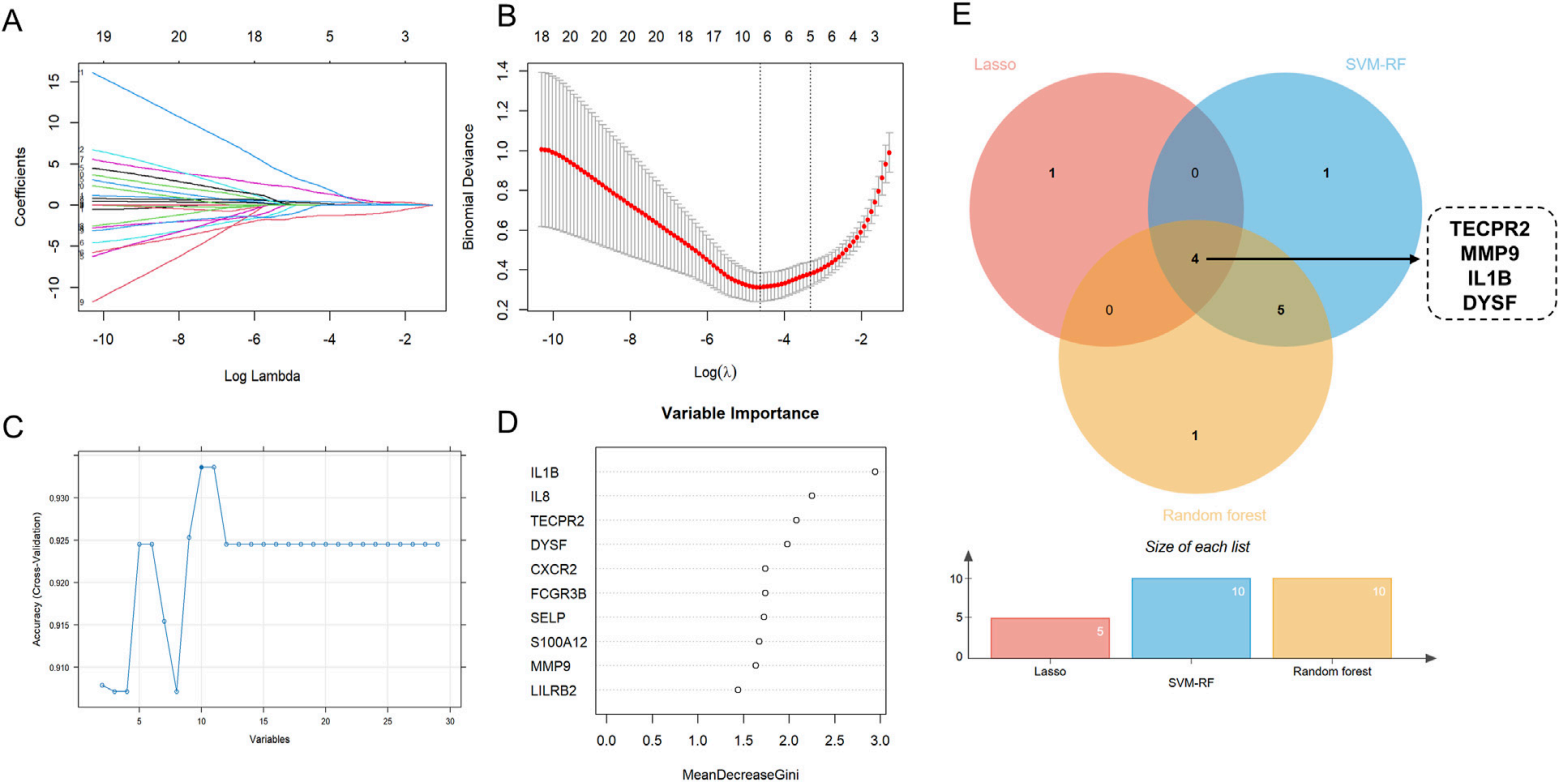
Identification Key DEONRGs in UC. **(A,B)** By LASSO logistic regression algorithm, with penalty parameter tuning conducted by 10-fold cross-validation, was used to select NETs-related features; **(C)** SVM-RFE algorithm to filter the 10 DEONRGs to identify the optimal combination of feature genes; **(D)** RF algorithm to screen the top 10 DEONRGs to identify the optimal combination of feature genes; **(E)** Venn diagram showing the overlap of key genes identified by LASSO, SVM-RFE and RF in UC. Four common hub genes (TECPR2, IL-1B, MMP and DYSF) were identified across three machine learning model. LASSO, Least Absolute Shrinkage and Selection Operator; SVM-RFE, Support Vector Machine-Recursive Feature Elimination; RF, Random Forest.

Feature gene selection was performed using LASSO regression, SVM-RFE and RF models. LASSO regression, combined with 10-fold cross-validation, facilitated automatic feature selection and optimization of the regularization parameters, aiming to minimize prediction error. The LASSO algorithm successfully identified 5 feature variables when the lambda value was minimized (Figures 4A,B). The SVM-RFE model identified ten key genes based on the maximum accuracy (Figure 4C). By increasing the number of decision trees in the random forest model, the prediction error gradually decreased, stabilizing at approximately 300 trees (Supplementary Figure 1C). The random forest model identified the 10 feature genes with variable importance greater than 1 when selecting the smallest cross-validation error (Figure 4D). As shown in Supplementary Table 4, the three machine learning models demonstrated pretty consistency and reliability. A Venn diagram was used to intersect the core genes identified by the three machine learning methods, revealing that IL1B, MMP9, DYSF and TECPR2 were considered core genes of NRGs in UC (Figure 4E).

### Validation of hub genes expression and diagnostic performance

**FIGURE 5:**
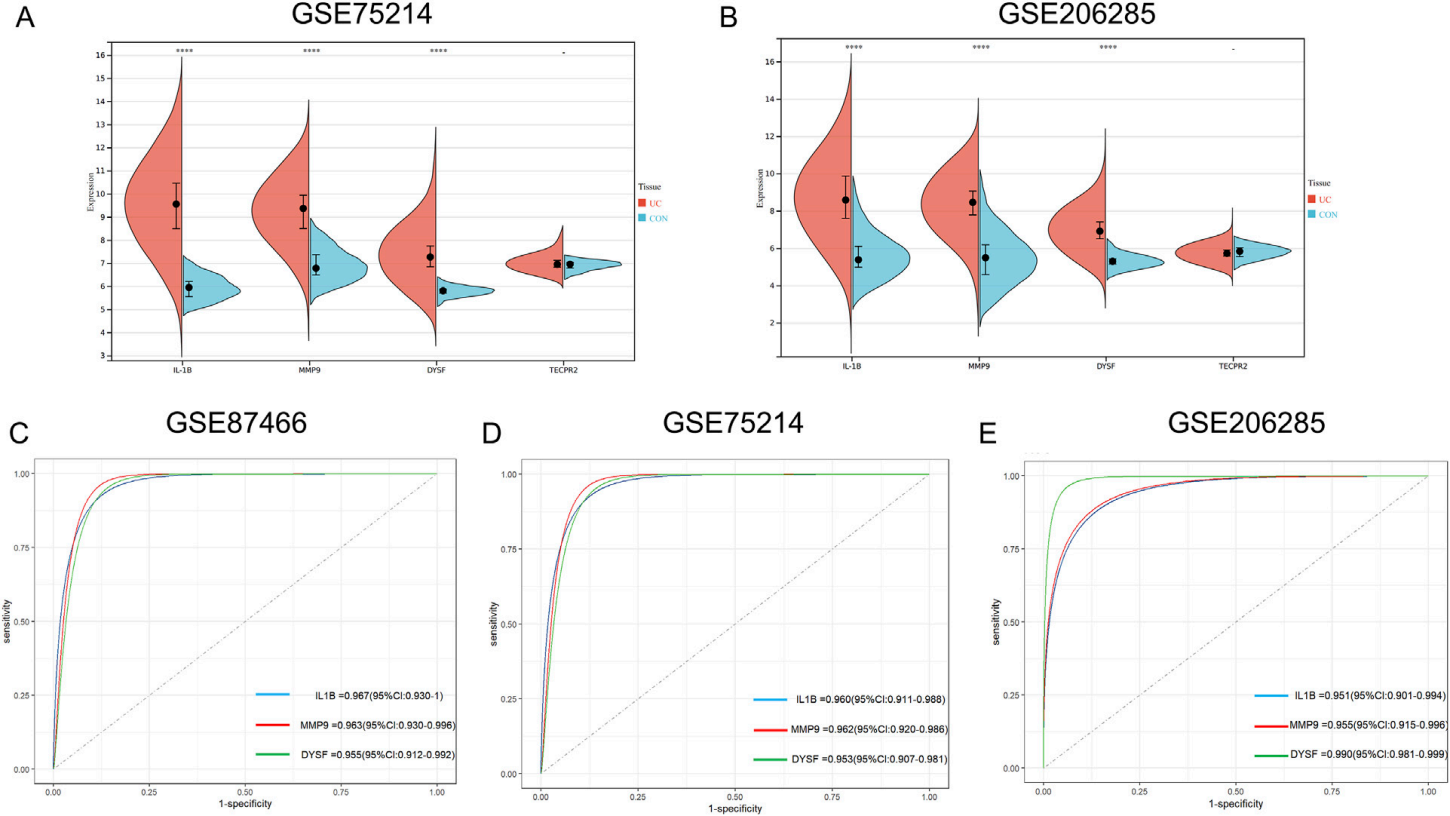
Identification the Expression and ROC Curves for Key Hub Genes in Training and Validation Sets for UC. **(A,B)** Box plot depicting the differential expression of the four candidate hub genes between UC patients and control groups in GSE75214 **(A)** and GSE206285 **(B) (C–E)** ROC curves for individual hub genes (IL-1B, MMP9 and DYSF) in GSE87466, with their corresponding AUC values **(C)** ROC curves for hub genes in the GSE75214 dataset, showing high predictive accuracy with AUC ranging from 0.953 to 0.962 **(D)** ROC curves for hub genes in the GSE206285 dataset, showing varying levels of predictive accuracy, with AUC values ranging from 0.951 to 0.990 **(E)** Statistical analysis was performed using unpaired t-tests. (*P < 0.05, **P < 0.01, ***P < 0.001) ROC, receiver operating characteristic; AUC, Area Under the Curve.

To validate our findings, we further confirmed the expression of the four genes in UC using external datasets, GSE75214 and GSE260258. The results showed that, except for TECPR2, IL1B, MMP9 and DYSF exhibited differential expression between UC and control groups (Figures 5A,B). Subsequently, we performed ROC curve analysis to explore the diagnostic performance of the core genes across three datasets. The results demonstrated that the AUC values for the core genes in all three datasets were greater than 0.9, indicating exceptional predictive ability. These core genes could therefore serve as key molecular biomarkers for diagnosing UC (Figures 5C–E).

### Gene set enrichment analysis

To further elucidate the relationship between core genes and the inflammatory and immune mechanisms involved in the pathogenesis of UC, we performed GSEA analysis based on the expression of core genes in the GSE87466 dataset. Based on the enrichment scores, we identified ten key signaling pathways related inflammation and immunity activated by DEONRGs in UC (Supplementary Figure 2). The expression of the three core genes was enriched in pathways related to NETs formation, immunity, and inflammation. The results indicated that high expression of IL-1B in UC was mainly associated with IL-17 signaling pathway, TNF signaling pathway, NF-kappa B signaling pathway and Viral protein interaction with cytokine and cytokine receptor. Elevated MMP9 expression was primarily linked to Primary immunodeficiency, Viral protein interaction with cytokine and cytokine receptor, NF-kappa B signaling pathway and Intestinal immune network for IgA production, while increased DYSF expression was mainly associated with Glycosaminoglycan biosynthesis-chondroitin sulfate dermatan sulfate, Primary immunodeficiency, Viral protein interaction with cytokine and cytokine receptor, ECM-receptor interaction and NF-kappa B signaling pathway. The nominal *p*-values, FDR q-values, and NES of the immunity and inflammation gene sets related to hub genes expression in GSE87466 are provided in Supplementary Table 5.

### Immune infiltration analysis

**FIGURE 6:**
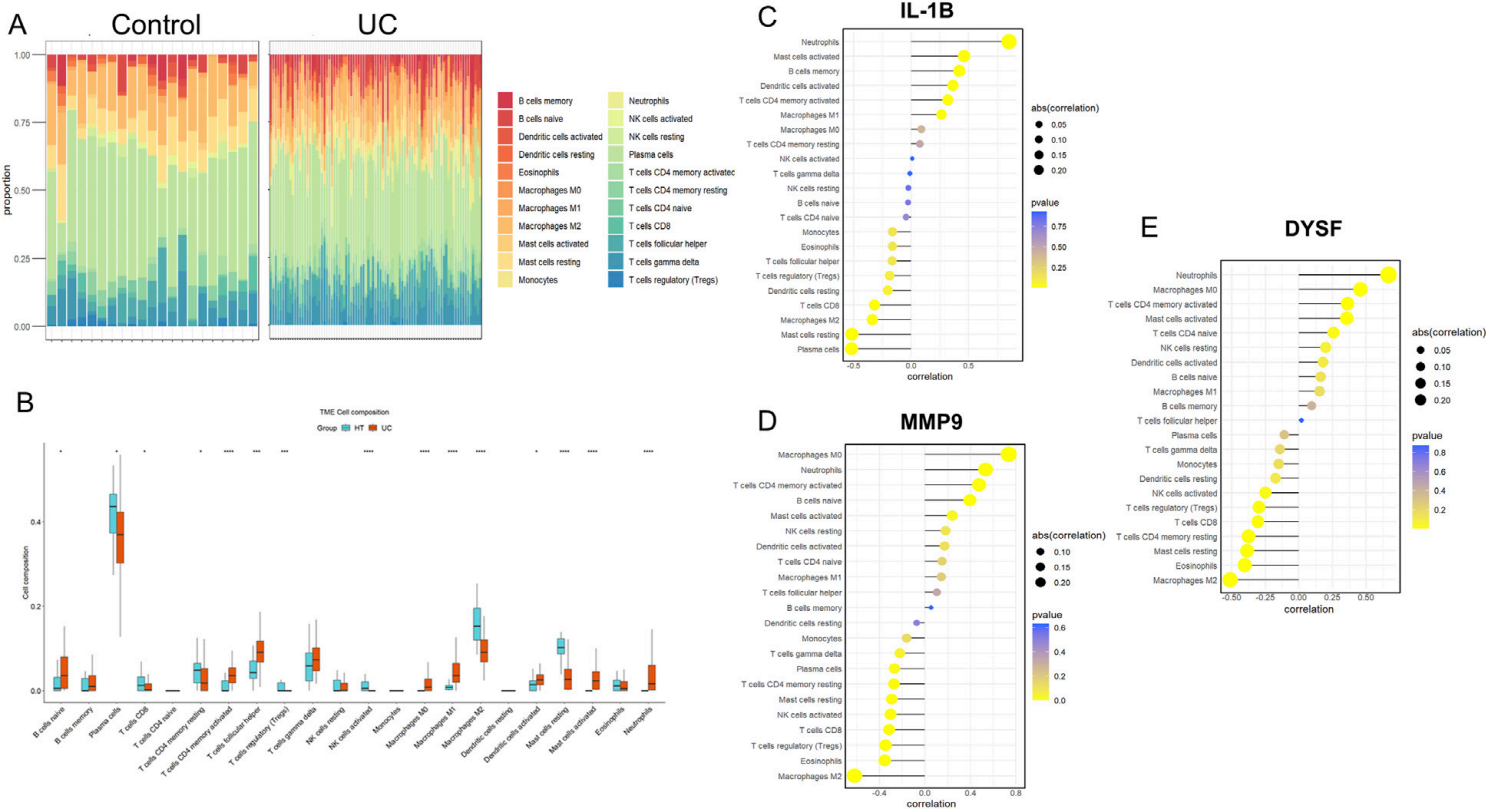
Immune Cell Infiltration Analysis for UC Datasets. Stacked proportional bar chart of immune cells between UC group and control group **(A)** Boxplots illustrating the proportions of various immune cell types in the control (blue) and disease (red) groups in the datasets GSE 87466. Significant differences in cell type abundance are observed between the groups. The *p*-values indicate the statistical significance of these differences between groups **(B)** The lollipop chart shows the results of correlation analysis between IL-1B **(C)** MMP9 **(D)** and DYSF **(E)** with various immune cell types in UC patients. The correlation coefficients are represented along with the *p*-values, indicating the strength and significance of the correlation between gene expression and immune cell infiltration. The size of the circles represents the absolute correlation (abs (cor)), and the color scale represents the *p*-value, with darker colors showing more significant correlations.

To further identify the immune cell types associated with UC in the colon, CIBERSORT was used to quantify the proportions of 22 immune cell types in normal colon and UC colon samples (Figure 6A). Compared to healthy controls, UC tissues exhibited a significant decrease in activated NK cells, regulatory T cells and M2 macrophages, while neutrophils, M0 macrophages, M1 macrophages, and CD4^+^ T memory cells were significantly increased (Figure 6B). We then performed Pearson correlation analysis to examine the relationship between these immune cells and the expression levels of core genes. The results (Table 1) revealed that the expression of the three core genes was significantly positively correlated with neutrophils infiltration (r > 0.5, p < 0.001). Furthermore, IL-1B was positively correlated with activated mast cell, memory B cells and dendritic cells, and negatively correlated with plasma cells and resting mast cells (Figure 6C). MMP9 was significantly positively correlated with M0 macrophages in UC, while significantly negatively correlated with M2 macrophages (Figure 6D). DYSF was positively correlated with M0 macrophages, activated CD4^+^ T memory cells, and mast cells, while negatively correlated with M2 macrophages and eosinophils (Figure 6E). These results suggest that immune responses mediated by NRGs play a crucial role in the pathogenesis of UC.

**TABLE 1:** The results of sperman analysis between core genes and immune cells.

Immune cells	IL-1B		MMP9		DYSF	
	*correlation*	*pvalue*	*correlation*	*pvalue*	*correlation*	*pvalue*
B cells naive	−0.03	0.80	0.40	<0.001	0.16	0.14
B cells memory	0.42	<0.001	0.05	0.63	0.09	0.39
Plasma cells	−0.52	<0.001	−0.27	0.01	−0.11	0.31
T cells CD8	−0.32	<0.001	−0.31	<0.001	−0.31	<0.001
T cells CD4 naive	−0.04	0.68	0.15	0.16	0.26	0.02
T cells CD4 memory resting	0.07	0.50	−0.27	0.01	−0.37	<0.001
T cells CD4 memory activated	0.32	<0.001	0.48	<0.001	0.36	<0.001
T cells follicular helper	−0.17	0.12	0.10	0.33	0.02	0.87
T cells regulatory (Tregs)	−0.19	0.08	−0.35	<0.001	−0.30	0.01
T cells gamma delta	−0.01	0.91	−0.22	0.04	−0.14	0.20
NK cells resting	−0.02	0.83	0.18	0.09	0.20	0.06
NK cells activated	0.01	0.93	−0.30	<0.001	−0.25	0.02
Monocytes	−0.16	0.13	−0.16	0.13	−0.15	0.16
Macrophages M0	0.09	0.41	0.74	<0.001	0.46	<0.001
Macrophages M1	0.27	0.01	0.14	0.19	0.15	0.16
Macrophages M2	−0.34	<0.001	−0.62	<0.001	−0.51	<0.001
Dendritic cells resting	−0.20	0.06	−0.07	0.50	−0.17	0.11
Dendritic cells activated	0.37	<0.001	0.17	0.11	0.18	0.10
Mast cells resting	−0.51	<0.001	−0.29	0.01	−0.39	<0.001
Mast cells activated	0.46	<0.001	0.24	0.03	0.36	<0.001
Eosinophils	−0.16	0.13	−0.35	<0.001	−0.40	<0.001
Neutrophils	0.85	<0.001	0.53	<0.001	0.67	<0.001

### Expression of hub genes in UC patients

**FIGURE 7:**
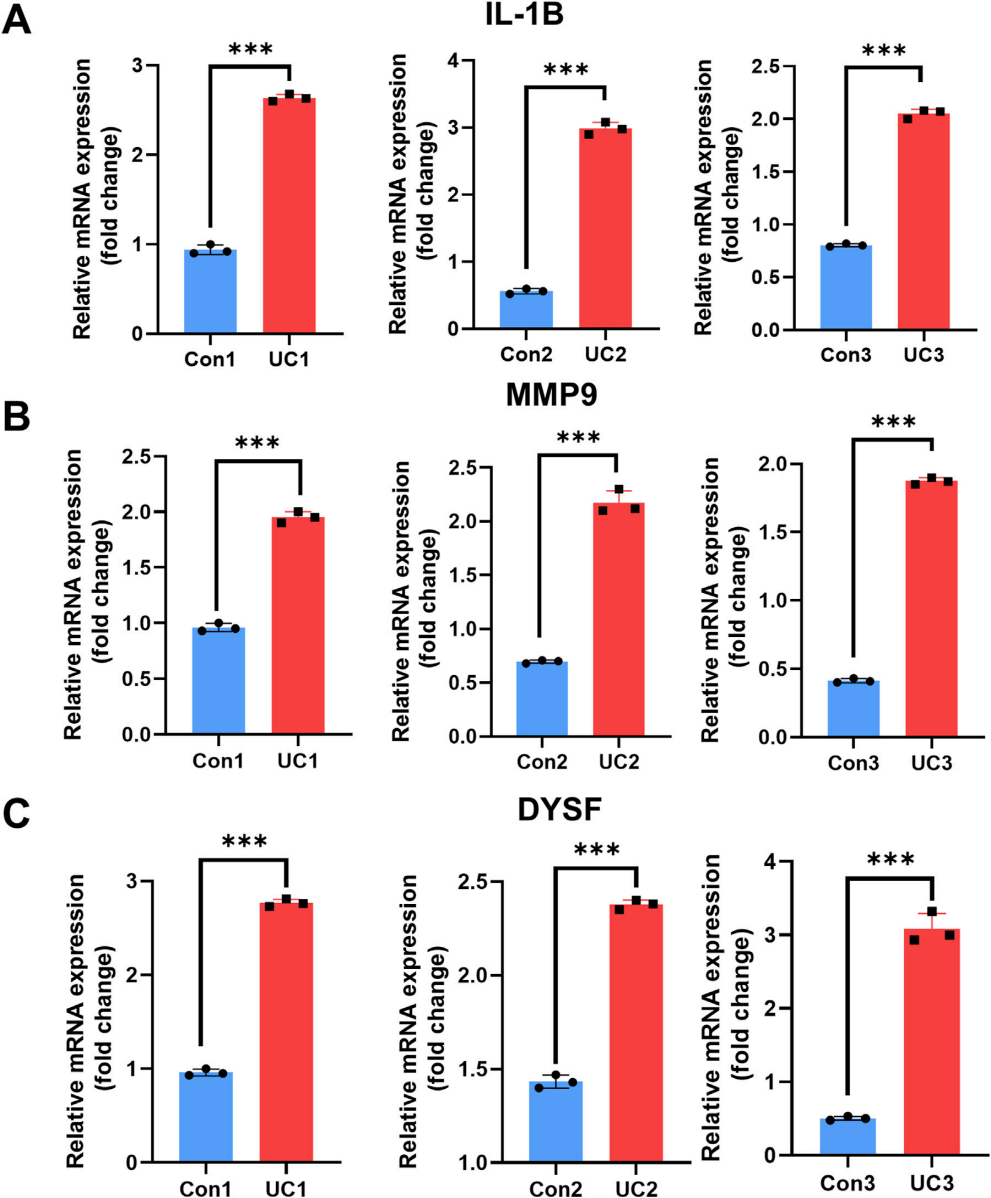
Expression analysis of IL-1B, MMP9 and DYSF in control and UC groups. **(A–C)** Quantitative qRT-PCR results showing mRNA expression levels of IL-1B **(A)** MMP9 **(B)** and DYSF **(C)** in three biological replicates from the control group (blue) and UC group (red). Data are presented as fold change relative to the control group. Statistical analysis was performed using unpaired t-tests (*P < 0.05, **P < 0.01, ***P < 0.001)

To further validate our hypothesis, we measured the transcriptional levels of IL-1B, MMP9, and DYSF in blood samples collected from UC patients (n = 3) and healthy individuals (n = 3) by qRT-PCR. All three genes showed a marked upregulation in the UC group, with statistically significant differences observed across three independent biological replicates (Figures 7A–C), (*P* < 0.05 to *P* < 0.001). These findings suggest that IL-1B, MMP9 and DYSF play a role as core NETs targets in the progression of UC, highlighting their potential as candidate diagnostic biomarkers for identifying and monitoring severe UC cases.

### Potential drugs targeting neutrophil extracellular traps genes

**FIGURE 8:**
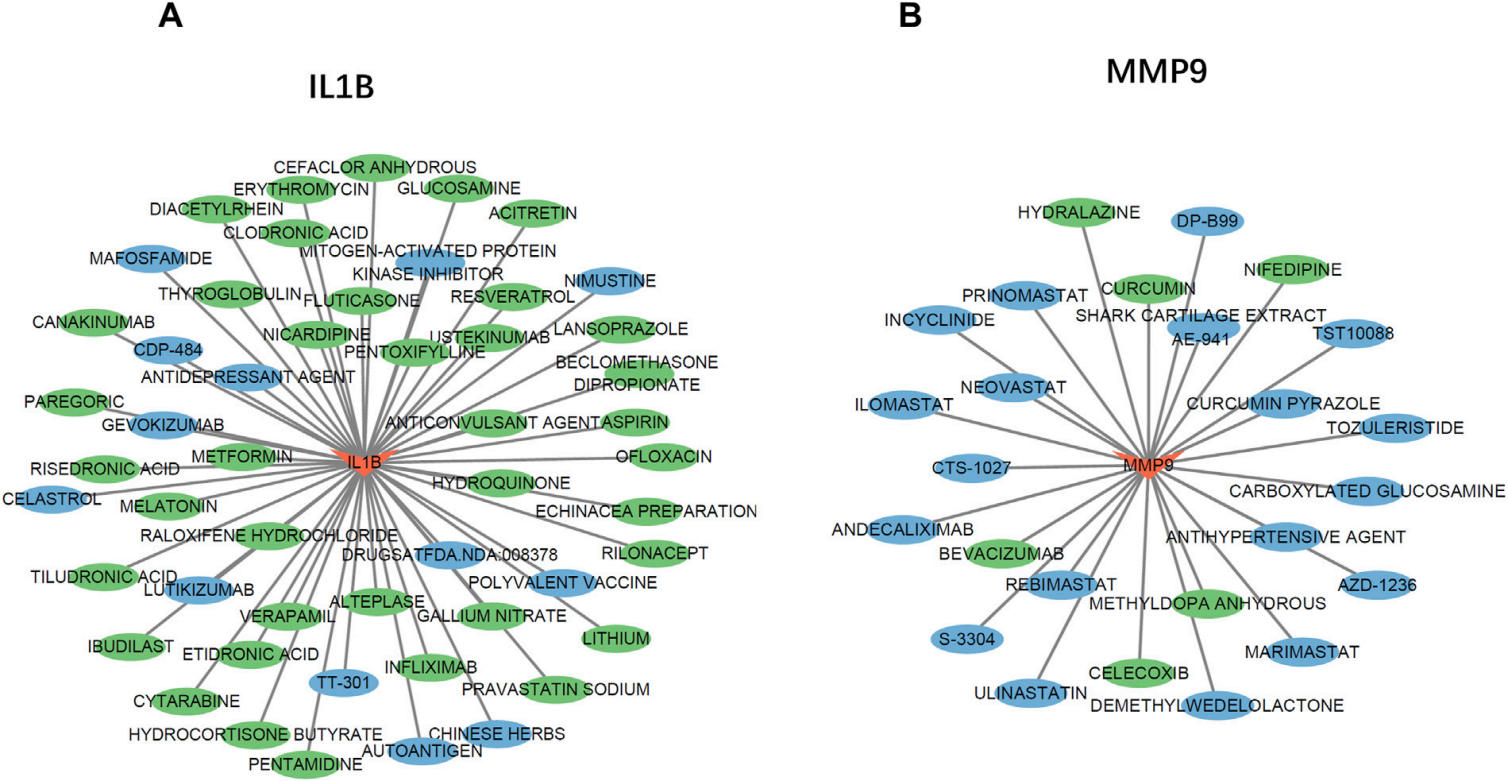
The Gene-Drug Network. **(A,B)** The drug-gene network of IL-1B **(A)** and MMP9 **(B)** Drugs marked in green have already undergone endometriosis-related clinical trials. Drugs marked in blue have already been tested on animal models.

To explore potential drugs for the treatment of UC, we searched the DGIdb database for drugs targeting NETs biomarkers. The drug–gene interaction results from the DGIdb database revealed 51 drugs targeting IL-1B and 25 drugs targeting MMP9. Regarding IL1B-targeting drugs, 37 have been approved for marketing, and 14 of these have undergone clinical trials related to UC (Figure 8A). The top five drugs with the highest interaction (IS) scores (IS > 2) are GEVOKIZUMAB, CANAKINUMAB, TT-301, PENTAMIDINE and POLYVALENT VACCINE. Among the MMP9-targeting drugs, six have been approved for marketing, and three have been proven effective in treating UC. Furthermore, three drugs have been shown to slow the progression of UC in animal studies (Figure 8B). The top five drugs with the highest IS (IS > 1) are CARBOXYLATED GLUCOSAMINE, ANDECALIXIMAB, DP-B99, ULINASTATIN, and CURCUMIN PYRAZOLE.

## Discussion

UC, a chronic inflammatory bowel disease characterized by mucosal immune dysregulation, has seen an increasing incidence in recent years. Over the past 3 decades, the prevalence of inflammatory bowel diseases (IBD) has risen by 47.45%, and this trend is expected to continue, with projections indicating that by 2030, IBD patients will comprise 1% of the global population (Wang S. et al., 2024). Despite the significant reduction in UC-related mortality due to the use of immunosuppressive drugs and biologics, delayed diagnosis and the individual variability in treatment response pose significant challenges in achieving complete remission for UC patients (Hirten and Sands, 2021). NETs have long been associated with the local immune responses and pathological processes in IBD. Excessive NETs release may impair intestinal barrier function, sustain inflammation, and exacerbate tissue damage and poor repair, further promoting the destruction of the intestinal barrier and contributing to chronic inflammation (Li et al., 2021). Several studies have previously demonstrated the role of neutrophils in the pathogenesis of UC. One study identified two neutrophil-associated gene subtypes and their biological functions involved in UC, but it did not further elucidate the core genes driving NETs formation and their specific roles in UC (Zhang et al., 2023). Another Mendelian study confirmed the involvement of NETs in UC from a genetic perspective (Xv et al., 2024). However, due to limitations related to specific populations and disease activity, the results of the Mendelian study require further comprehensive bioinformatics analysis to clarify the biological functions and mechanisms of NETs, particularly in UC, a disease influenced by multiple factors including genetics, environment, and immunity.

This study aimed to better understand the specific role of NETs in UC through bioinformatics and machine learning approaches, identifying potential diagnostic and therapeutic targets. We used the limma and WGCNA packages for comparative analysis and to identify DEGs. A Venn diagram was then used to obtain overlapping NRGs from the DEGs, followed by comprehensive enrichment analysis to explore the biological pathways associated with these NRGs. The analysis revealed that DEGs were primarily enriched in “leukocyte migration activation”,“immune receptor activity” and the IL-17 signaling pathway. Next, we constructed a PPI network of NRGs using the STRING database, revealing strong interconnections among the NRGs in UC. To further identify core genes within the DEONRGs, we applied three machine learning methods, resulting in the selection of four candidate core genes: IL-1B, MMP9, DYSF and TECPR2. After validation using external datasets, IL-1B, MMP9 and DYSF were confirmed as core NRGs. Excellent ROC curve performance further validated their potential as biomarkers for early UC diagnosis. Immune infiltration analysis were conducted to determine the immune cell functions associated with the core NRGs. The results of qRT-PCR further validated our findings, and the potential therapeutic drugs were identified using the DGIdb database.

IL-1B is a factor synthesized and secreted by macrophages, monocytes, and other cell types, belonging to the interleukin-1 (IL-1) family. It participates in various cellular activities, including cell proliferation, differentiation, and programmed cell death, by binding to its receptor IL-1R (Aschenbrenner et al., 2021). Previous studies have shown that IL-1B-induced NETs formation can promote experimental abdominal aortic aneurysm (Meher et al., 2018). In UC-related studies, targeting IL-1B effectively alleviates colitis in mice (Cook et al., 2013). Our immune infiltration and GSEA analysis results suggest that, aside from neutrophils, IL-1B is strongly correlated with B cells memory and dendritic cells activated. We suggest the high expression of IL-1B may activate dendritic cells through NF-kappa B signaling pathway, leading to the secretion of IL-1 and TNF, which subsequently activate NETs (Chen et al., 2024b). In addition, the expression of IL-1B may active the memory B cells, continuous activation of memory B cells may lead to the loss of intestinal immune tolerance, resulting in abnormal activation of the intestinal immune system and attacking self-tissues through the interaction between memory B cells and the IL-17 signaling pathway (He et al., 2024). Furthermore, viral protein interaction with cytokine and cytokine receptor may activate mast cells through the Toll-like receptor signaling pathway, leading to increased IL-1 secretion, enhanced vascular permeability at the secretion site, and subsequent migration of white blood cells to the inflammatory site (Gebremeskel et al., 2021). This creates persistent inflammation that contributes to the development of UC.

Matrix metalloproteinase-9 (MMP9), a member of the zinc-dependent endopeptidase family, plays a crucial role in immune activation, inflammation cascade regulation, and extracellular matrix degradation and remodeling. MMP9 facilitates the accumulation of immune cells in the pathogenesis of various diseases (Mei et al., 2022). Observational studies have reported high expression of MMP9 in the inflamed mucosal regions of UC (B et al., 1999). Multiple studies have confirmed that MMP9 induces tissue damage via the NETs pathway in diseases such as osteoarthritis and myocardial infarction (Ke et al., 2024; Luan et al., 2023). Our study provides additional insights into the mechanism of MMP9-mediated NETs damage in UC. Immune infiltration analysis shows that the expression of MMP9 is significantly correlated with the infiltration of M0 macrophages and CD4^+^ T cells in UC. GSEA enrichment analysis suggests that MMP9 may promote the polarization of M0 macrophages into M1 macrophages through the NF-κB and TLR signaling pathways, leading to the secretion of pro-inflammatory cytokines such as TNF-α,IL-1β, and IL-6, which then contribute to NETs formation and mucosal damage (Pucci et al., 2021). Additionally, the correlation between MMP9 high expression and CD4 T cell infiltration suggests that MMP9 may active CD4^+^ memory T cells, promoting the Th17 cell differentiation and release of pro-inflammatory cytokines such as IFN-γ, IL-17, and TNF-α, which enhance NETs formation and excessive immune responses (Jiang et al., 2023). Meanwhile, NETs and their histones further promote Th17 cell differentiation directly via TLR2, ultimately leading to chronic inflammation and tissue damage (Wilson et al., 2022).

Currently, there is no evidence supporting the use of dysferlin (DYSF) as a biomarker for UC. However, dysregulation of DYSF expression is closely associated with various hereditary myopathies and autoimmune diseases. For example, upregulation of DYSF expression plays a key role in inflammatory cell infiltration and muscle damage in dermatomyositis and idiopathic inflammatory myopathy (Xiao et al., 2019). Moreover, DYSF promotes monocyte activation, enhancing its phagocytosis, adhesion, and migration, thus contributing to the formation of necrotic cores in atherosclerosis and playing an important role in atherosclerotic cardiovascular disease. It has been confirmed as a core diagnostic biomarker for atherosclerosis and systemic lupus erythematosus (Ding et al., 2023; Zhang X. et al., 2022). In our study, DYSF demonstrated excellent discriminatory ability, suggesting its potential as a candidate biomarker for UC. Combining previous research with our immune infiltration results, we found that DYSF may mediate NETs formation through both intestinal mucosal barrier and immune cell activation, promoting the progression of UC. On the one hand, DYSF is significantly correlated with CD4 T cell infiltration in UC, which may promote Th1 and Th2 cell differentiation, leading to the production of cytokines such as IFN-γ, IL-4, IL-5, IL-13, and TNF-α (Gomez-Bris et al., 2023). Activated Th1 cells secrete IFN-γ and TNF-α, recruiting and activating neutrophils, which enhances NETs formation. On the other hand, our enrichment analysis revealed that DYSF expression is associated with mucosal barrier pathways, including ECM-receptor interaction and glycosaminoglycan biosynthesis, specifically chondroitin sulfate/dermatan sulfate pathways in UC (Long et al., 2024). This suggests that DYSF may affect the repair and regeneration of intestinal epithelial cells via these pathways. Inadequate repair of the damaged intestinal mucosal barrier leads to the excessive activation of immune cells and the recruitment of pro-inflammatory factors, triggering NETs formation and further damage to intestinal tissue.

In our study, we have, for the first time, identified and validated the core NETs genes in UC, exploring their molecular functions, signaling pathways, and immune-mediated actions, and screened potential therapeutic drugs for UC based on these core genes. Despite our efforts to improve the reliability of the findings by utilizing large datasets, multiple analytical methods, and both internal and external validation, there are inevitable limitations in our research. Firstly, our samples were derived from previously published datasets, potential sample bias and limited representativeness may compromise the generalizability of the findings, and variations in dataset selection and analytical methods could lead to different outcomes. Secondly, as the study of NETs deepens, the gene set associated with NETs requires further refinement. Lastly, the lack of additional molecular experiments or animal studies limits our understanding of the mechanistic role of core genes in UC. Therefore, further experimental studies are necessary to confirm our findings.

## Conclusion

In conclusion, IL-1B, MMP9 and DYSF have been identified as core genes associated with UC-related NETs and are involved in the regulation of the immune microenvironment in UC. Our future research will focus on these genes in order to further elucidate the pathogenesis and management of UC. NETs-based approaches in UC management may contribute to its potential for complete cure in the future.

## Data Availability

The original contributions presented in the study are included in the article/Supplementary Material, further inquiries can be directed to the corresponding author.

## Supplementary material

SUPPLEMENTARY FIGURE S1 Scatterplot of MM and GS from the salmon module in UC **(A)** PPI network diagram **(B)** Decision tree of RF model in GSE87466 **(C)** Abbreviations: UC: ulcerative colitis; GS, Gene Significance; MM, Module Membership; PPI: protein-protein interaction. RF: Random forest.

SUPPLEMENTARY FIGURE S2 An integrated gene set enrichment analysis plot of DYSF **(A)** IL-1B **(B)** and MMP9 **(C)** in dataset GSE87466.

## Abbreviations

UC, Ulcerative colitis; NETs, Neutrophil Extracellular Traps; GEO, Gene Expression Omnibus; NRGs, Neutrophil extracellular traps related genes; DEGs, Differentially expressed genes; WGCNA Weighted gene co-expression network analysis; DEONRGs, Differentially expressed genes related to neutrophil extracellular traps; DYSF, Dysferlin; IL-1B, Interleukin-1 Beta; MMP9, Matrix Metalloproteinase-9; qRT-PCR, Quantitative Real-Time Polymerase Chain Reaction; IBD, Inflammatory bowel disease; GO, Gene Ontology; KEGG, Kyoto Encyclopedia of Genes and Genomes; BP, Biological Process; CC, Cellular Component; MF Molecular Function; PPI, Protein-protein interaction; MCODE, Molecular complex detection technology; LASSO, Least Absolute Shrinkage and Selection Operator; SVM-RFE, Support Vector Machine-Recursive Feature Elimination; RF, Random Forest; ROC, Receiver operating characteristic; AUC, Area Under the Curve; GSEA, Gene Set Enrichment Analysis; NES, Normalized enrichment score; FDR, False positive rate; IS, Interaction scores.
