# Supplementary material for: Identification of neutrophil extracellular trap-related biomarkers in ulcerative colitis based on bioinformatics and machine learning
Source: Front Genet. 2025 Jun 20;16:1589999. doi: 10.3389/fgene.2025.1589999 (PMC12226468; doi:10.3389/fgene.2025.1589999)
Supplement: Supplementary file 1 [file Table1.docx]

**Supplementary Table 1**. Details of samples and genes of all datasets in this study.

| Data sets | PMID | Number of genes | Healthy samples | Patients’ samples |
| --- | --- | --- | --- | --- |
| GSE87466 | PMID:29401083 | 20634 | 21 | 87 UC samples |
| GSE75214 | PMID:28885228 | 20001 | 11 | 97 UC samples |
| GSE206285 | PMID:36192482 | 21597 | 18 | 550 UC samples |

**Supplementary Table 2.** Information of 69 neutrophil extracellular traps related genes collected in this research.

| **Gene symbol** | | | |
| --- | --- | --- | --- |
| ALPL | TECPR2 | ITGAM | VNN3 |
| BST1 | TNFRSF10C | ITGB2 | MMP9 |
| CD93 | AKT1 | KCNN3 | KCNJ15 |
| CREB5 | AKT2 | MAPK1 | PADI4 |
| CRISPLD2 | ATG7 | MAPK3 | FCGR3B |
| CSF3R | CLEC6A | MTOR | S100A12 |
| DYSF | CSF3 | PTAFR | CEACAM3 |
| CPPED1 | CTSG | PIK3CA | FPR2 |
| FPR1 | CYBB | RIPK1 | CXCR2 |
| G0S2 | DNASE1 | RIPK3 | FCAR |
| HIST1H2BC | ELANE | SELP | CYP4F3 |
| HPSE | ENTPD4 | SELPLG | MGAM |
| CXCR1 | F3 | SIGLEC14 |  |
| LILRB2 | HMGB1 | TLR2 |  |
| MME | IL17A | TLR4 |  |
| PDE4B | IL1B | TLR7 |  |
| SIGLEC5 | IL6 | TLR8 |  |
| SLC22A4 | IL8 | TNF |  |
| SLC25A37 | IRAK4 | MPO |  |

**Supplementary Table 3**: The sequences of the primers for qRT-PCR.

| **Genes** | **Forword** | **Reverse** |
| --- | --- | --- |
| **IL-1B** | ACGATGCACCTGTACGATCACT | GAGAACACCACTTGTTGCTCCA |
| **MMP9** | TCGAACTTTGACAGCGACAAG | TCAGTGAAGCGGTACATAGGGT |
| **DYSF** | CAGAAGATCCATCTGTGATTGGT | CAATGTAGATACGGACCAAGCACT |

**Supplementary Table 4**: Evaluation parameters for three machine learning models.

| **Model** | **LASSO** | **SVM-RFE** | **RF** |
| --- | --- | --- | --- |
| Accuracy | 0.954 (0.895- 0.985) | 0.905 (0.696 - 0.988) | 0.952 (0.762- 0.998) |
| Precision | 0.966 | 0.941 | 1.000 |
| Recall | 0.977 | 0.940 | 0.941 |
| F1 score | 0.971 | 0.941 | 0.970 |
| AUC | 0.986 (0.969-1.000) | 0.971 ( 0.904-1.000) | 0.985 (0.945-1.000) |

**Supplementary Table 5:** The p-values, q-values, and NES of the gene sets related to immunity and inflammation of hub genes in GSE75214

| Gene-sets | IL-1B | | |  | MMP9 | | |  | DYSF | | |  |
| --- | --- | --- | --- | --- | --- | --- | --- | --- | --- | --- | --- | --- |
|  | p-val | FDR q-val | NES | Enrichment score | p-val | FDR q-val | NES | Enrichment score | p-val | FDR  q-val | NES | Enrichment score |
| IL-17 signaling pathway | <0.001 | <0.001 | 2.387 | 0.727 | <0.001 | <0.001 | 2.089 | 0.633 | <0.001 | 0.001 | 2.039 | 0.604 |
| Intestinal immune network for IgA production | <0.001 | <0.001 | 1.870 | 0.675 | <0.001 | <0.001 | 1.900 | 0.688 | - | - | - | - |
| TNF signaling pathway | <0.001 | <0.001 | 2.385 | 0.705 | <0.001 | <0.001 | 2.010 | 0.591 | - | - | - | - |
| NF-kappa B signaling pathway | <0.001 | <0.001 | 2.385 | 0.705 | <0.001 | <0.001 | 2.321 | 0.700 | <0.001 | 0.001 | 2.129 | 0.627 |
| Viral protein interaction with cytokine and cytokine receptor | <0.001 | <0.001 | 2.477 | 0.758 | <0.001 | <0.001 | 2.365 | 0.720 | <0.001 | 0.001 | 2.338 | 0.696 |
| B cell receptor signaling pathway | - | - | - | - | <0.001 | <0.001 | 2.198 | 0.677 | <0.001 | 0.001 | 2.024 | 0.610 |
| Th17 cell differentiation | <0.001 | <0.001 | 2.018 | 0.612 | <0.001 | <0.001 | 2.116 | 0.640 | <0.001 | 0.001 | 1.960 | 0.578 |
| Th1 and Th2 cell differentiation | - | - | - | - | <0.001 | <0.001 | 2.053 | 0.639 | <0.001 | 0.001 | 1.973 | 0.599 |
| Primary immunodeficiency |  |  |  |  | <0.001 | <0.001 | 2.273 | 0.809 | <0.001 | 0.001 | 2.141 | 0.742 |
| Neutrophil extracellular trap formation | <0.001 | <0.001 | 2.127 | 0.635 | <0.001 | <0.001 | 1.968 | 0.585 | <0.001 | 0.001 | 1.964 | 0.570 |
