# Supplementary figures and images for: Identification of neutrophil extracellular trap-related biomarkers in ulcerative colitis based on bioinformatics and machine learning
Source: Front Genet. 2025 Jun 20;16:1589999. doi: 10.3389/fgene.2025.1589999 (PMC12226468; doi:10.3389/fgene.2025.1589999)

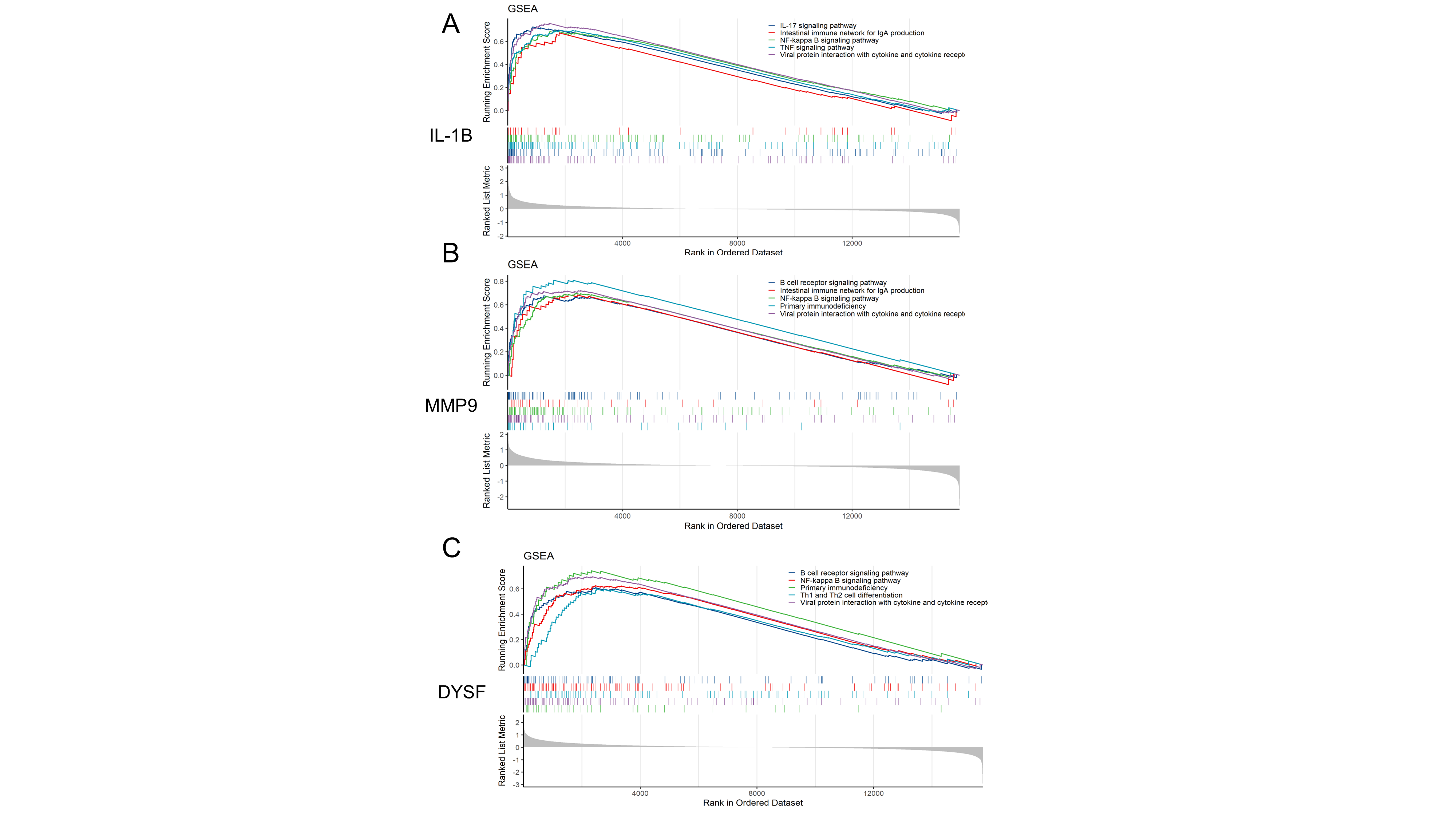

Supplement: Supplementary file 2 [file Image2.tif]

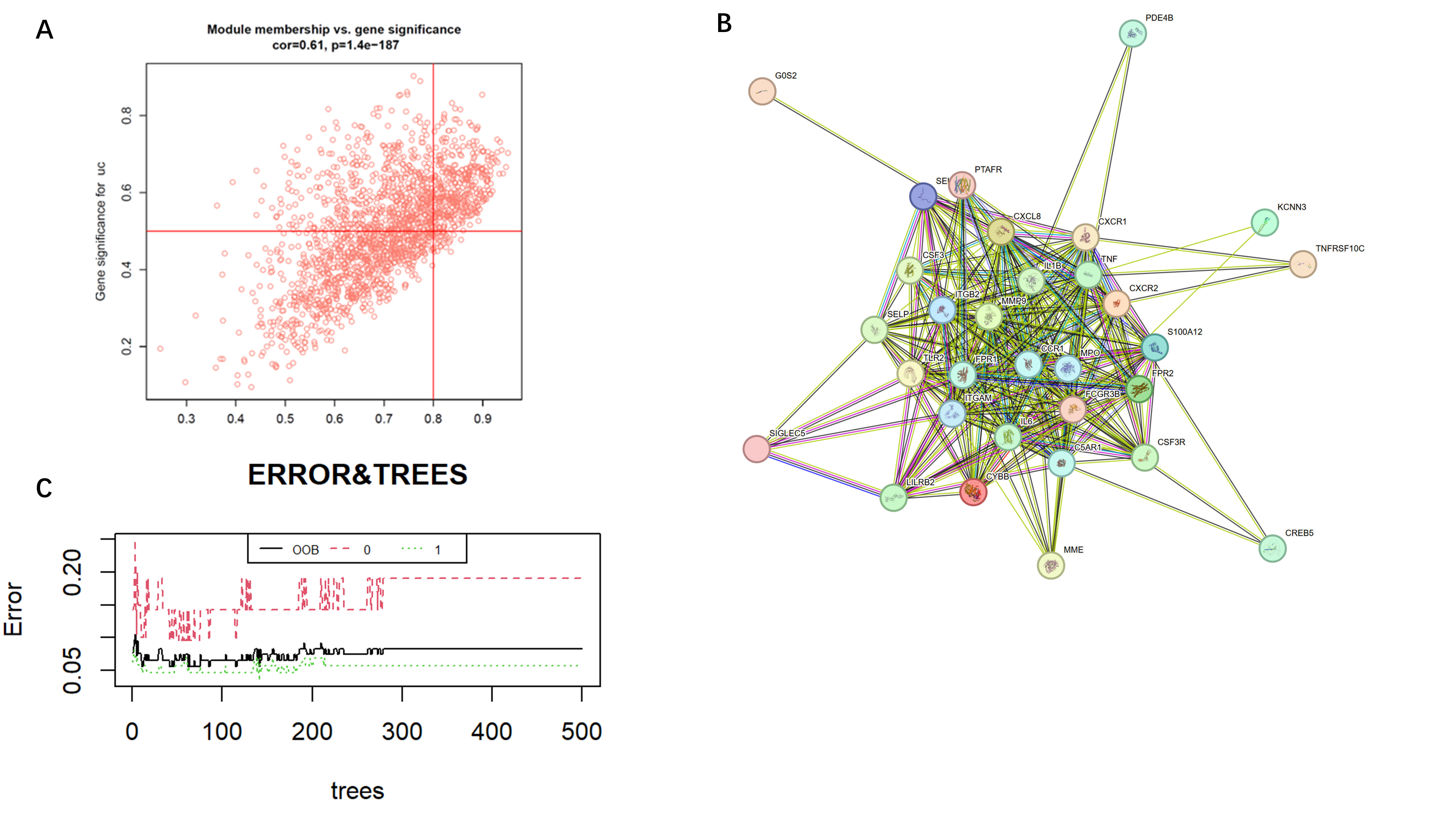

Supplement: Supplementary file 3 [file Image1.tif]
